# Therapeutic Potential of BMX-001 for Preventing Chemotherapy-Induced Peripheral Neuropathic Pain

**DOI:** 10.3390/ph18081159

**Published:** 2025-08-05

**Authors:** Tianshu Pan, Olawale A. Alimi, Bo Liu, Mena A. Krishnan, Mitchell Kuss, Wei Shi, Jairam Krishnamurthy, Jianghu James Dong, Rebecca E. Oberley-Deegan, Bin Duan

**Affiliations:** 1 Mary & Dick Holland Regenerative Medicine Program, University of Nebraska Medical Center, Omaha, NE 68198, USA; tpan@unmc.edu (T.P.); oalimi@unmc.edu (O.A.A.); bo.liu@unmc.edu (B.L.); mekrishnan@unmc.edu (M.A.K.); mitchell.kuss@unmc.edu (M.K.); weshi@unmc.edu (W.S.); 2 Division of Cardiology, Department of Internal Medicine, University of Nebraska Medical Center, Omaha, NE 68198, USA; 3 Department of Biochemistry and Molecular Biology, University of Nebraska Medical Center, Omaha, NE 68198, USA; 4 Division of Oncology & Hematology, Department of Internal Medicine, University of Nebraska Medical Center, Omaha, NE 68198, USA; jairam.krishnamurthy@hsc.utah.edu; 5 Department of Biostatistics, University of Nebraska Medical Center, Omaha, NE 68198, USA; jianghu.dong@unmc.edu; 6 Department of Surgery, University of Nebraska Medical Center, Omaha, NE 68198, USA; 7 Department of Mechanical and Materials Engineering, University of Nebraska-Lincoln, Lincoln, NE 68588, USA

**Keywords:** neuropathy, BMX-001, paclitaxel, satellite glial cells, reactive oxygen species

## Abstract

**Background/Objectives**: Chemotherapy-induced neuropathic pain (CINP) represents a critical challenge in oncology, emerging as a common and debilitating side effect of widely used chemotherapeutic agents, such as paclitaxel (PTX). Current therapeutic interventions and preventive strategies for CINP are largely insufficient, as they fail to address the underlying peripheral nerve damage, highlighting an urgent need for the development of new drugs. This study aimed to investigate the dual-function effects on normal cell protection and tumor suppression of BMX-001, a redox-active manganese metalloporphyrin that has demonstrated antioxidant and anti-inflammatory properties, which offers potential in protecting central nervous system tissues and treating CINP. **Methods**: This study assessed BMX-001’s different roles in protecting normal cells while acting as a pro-oxidant and pro-inflammatory molecule in cancer cells in vitro. We also evaluated its neuroprotective effect in preclinical PTX-induced CINP models in vivo. **Results**: Our results showed significant reductions in mechanical and cold allodynia, decreased pro-inflammatory cytokine levels, and restored antioxidant capacity in peripheral nerves and dorsal root ganglia (DRGs) following BMX-001 treatment. **Conclusions**: Overall, our study highlights the therapeutic potential of BMX-001 to mitigate CINP and enhance anticancer efficiency. Its dual-selective mechanism supports the future clinical investigation of BMX-001 as a novel adjunct to chemotherapeutic regimens.

## 1. Introduction

Chemotherapy-induced neuropathic pain (CINP) remains a significant dose-limiting toxicity associated with widely used chemotherapeutic agents, such as paclitaxel (PTX) [1]. Rather than arising from metabolic dysfunction or mechanical injury, like diabetic or traumatic neuropathy, CINP is triggered directly by neurotoxic chemotherapeutic agents, like taxanes and platinum and typically follows a bilateral “glove and stocking” distribution during the chemotherapy treatment cycles and could persist for months or years after therapy ends [2,3]. Although PTX is a standard treatment for many malignancies, such as breast, ovarian, and endometrial cancers, due to its potent antitumor activity, it often induces progressive neuropathy in patients, which limits its overall effectiveness [4,5]. In approximately 30% of patients, CINP presents persistent sensory disorders, including pain, tingling, and numbness, which significantly reduce quality of life and interfere with treatment plans [6,7]. Although topical agents like capsaicin, antidepressants like duloxetine, and anticonvulsants such as gabapentin and pregabalin are currently used to manage CINP, these treatments primarily focus on symptom control rather than preventing the underlying neuronal injury [8,9]. Their effectiveness is generally limited due to the development of multiple adverse effects, such as drowsiness, dizziness, and gastrointestinal problems, which further diminish patients’ quality of life [10,11]. Currently, no effective therapies exist to address the underlying neuropathic damage in CINP, highlighting the urgent need for novel interventions that target neuronal injury and pain sensitization.

The underlying mechanisms of CINP are multifactorial, with oxidative stress and neuroinflammation playing critical roles [12]. Chemotherapeutic agents, including platinum compounds, taxanes, and vinca alkaloids, have been shown to induce excessive production of reactive oxygen species (ROS), thereby damaging the peripheral neurons [13,14]. As a taxane, PTX causes nerve damage and neurotoxicity by binding to β-tubulin and preventing the disassembly of neuronal microtubules [15]. This disruption impairs axonal transport along the long projections of neurons, increasing their vulnerability to the microtubule-stabilizing effects of PTX [16]. The resulting transport deficits lead to local ATP insufficiency and disrupted calcium homeostasis, which together cause synaptic dysfunction, mitochondrial damage, ROS accumulation, and activation of glial and immune cells, compounding the neuroinflammatory response [17].

Emerging data suggests that anti-inflammatory and antioxidant components may help alleviate CINP symptoms [18]. Several such agents have been investigated under preclinical and clinical settings, including the clinically recommended duloxetine, which modulates key pathways involved in oxidative stress and neuroinflammation [19], and acetyl-L-carnitine, which has shown neuroprotective and antioxidant effects following chemotherapy in clinical trials [20]. However, these drugs often suffer from poor target specificity, limited efficacy, and notably, potential tumor-promoting effects, which restrict their broader clinical use [21,22]. Despite the growing interest in antioxidant and anti-inflammatory approaches for treating CINP, there remains a critical gap in identifying agents that simultaneously mitigate neuronal damage caused by ROS accumulation and inflammation while preserving chemotherapeutic efficacy.

BMX-001, a novel manganese metalloporphyrin initially studied for its role in mitigating damage induced by radiation therapy, offers potential as a therapeutic for preventing the underlying mechanisms of CINP [23]. Acting as a superoxide dismutase mimetic, BMX-001 has been reported to protect against radiation damage and modulate key transcription factors involved in oxidative stress and inflammation, for instance, inhibiting NF-κB and activating nuclear factor erythroid 2-related factor 2 (Nrf2) pathways [24,25]. Clinical studies have shown that BMX-001 can reduce radiation-related adverse effects, such as dermatitis and oral mucositis, by protecting healthy tissues from oxidative damage without sacrificing its anticancer efficacy [26,27]. In intestinal epithelial cells, BMX-001 has been shown to inhibit NF-κB and activate Nrf2, thereby regulating the expression of downstream pro-inflammatory cytokines and antioxidant enzymes following radiation therapy [28]. In addition, in brain endothelial cells, BMX-001 increased the expression of cytoprotective genes, such as HO-1 and NQO1, which are under the control of Nrf2 [29]. Interestingly, BMX-001 appears to have differential effects in normal versus cancer cells. According to a previous study, while BMX-001 reduced ROS in normal tissue with Nrf2 activation and NF-κB inhibition, it did not confer the same protective effects in cancer cells, including glioblastoma cell lines [23].

In healthy cells, BMX-001 functions as an MnSOD mimetic that reduces superoxide and activates antioxidant responses through regulating the redox state and inflammation through Nrf2 [30,31]. In tumor cells, where the ROS levels are already highly elevated and antioxidant defenses are often dysregulated and overloaded, Mn porphyrins like BMX-001 cause excess H_2_O_2_ accumulation and lead to increased tumor cell death [31,32]. This selective effect is likely due to the poor H_2_O_2_ detox capacity of cancer cells and altered redox signaling pathways [33]. Preclinical studies have also demonstrated that BMX-001 not only reduces neuroinflammation but also exhibits significant anticancer effects, including the inhibition of tumor proliferation and modulation of inflammatory cytokine activity [34,35], suggesting a possible role for BMX-001 in addressing CINP. Although BMX-001 can reduce ROS levels and inflammatory cytokine expression in peripheral nerves and dorsal root ganglia (DRGs), its exact efficacy in CINP prevention and differential effects on normal cells versus cancer cells require further validation.

In this study, we tested the central hypothesis that BMX-001 exerts cell-type-specific effects of acting as a protective antioxidant in healthy glial cells while enhancing oxidative stress in cancer cells to prevent CINP without affecting paclitaxel’s anticancer efficacy. We investigated the neuroprotective effects of BMX-001 in a preclinical mouse model of PTX-induced CINP. Through behavioral tests and histological evaluations, we demonstrated that BMX-001 mitigated oxidative stress, modulated inflammatory cytokine levels, and alleviated neuropathic pain sensitization. In summary, these findings support BMX-001 as a promising adjunct in chemotherapy protocols for preventing CINP and underscore the need for future clinical studies to evaluate its therapeutic potential.

## 2. Results

This section may be divided by subheadings. It should provide a concise and precise description of the experimental results, their interpretation, as well as the experimental conclusions that can be drawn.

### 2.1. BMX-001 Reduced Total ROS Elevated by PTX in Satellite Glial Cells (SGCs) but Increased ROS in MDA-MB-231 Cells In Vitro

**Figure 1:**
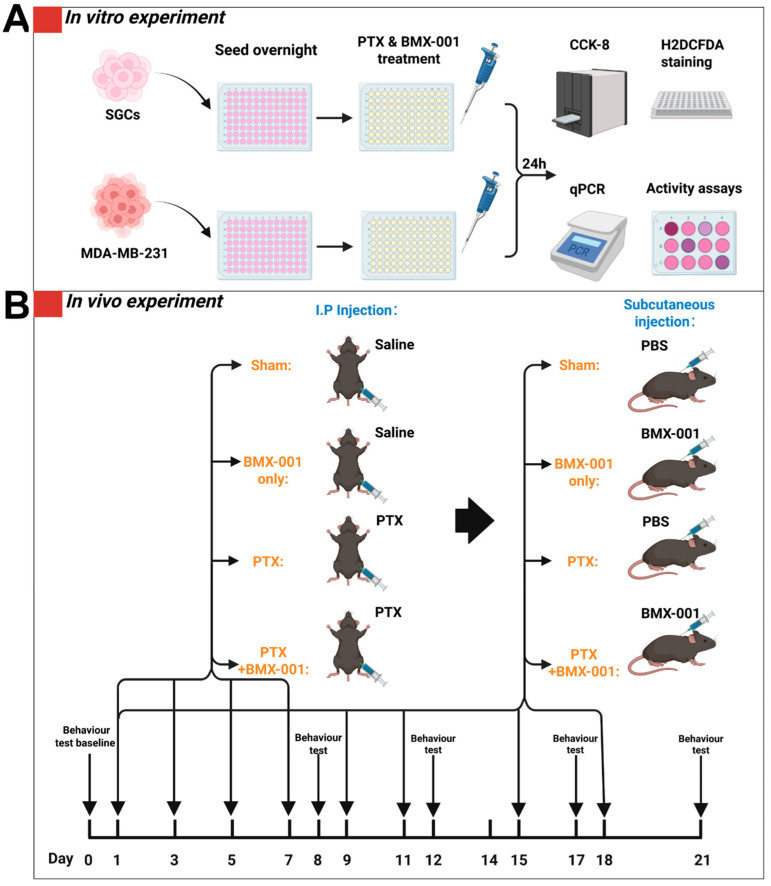
Schematic of the in vitro and in vivo experimental designs. (**A**) For in vitro experiments, SGCs and MDA-MB-231 cells were seeded in 96-well plates and cultured overnight in DMEM with 10% FBS and 1% P/S. The cells were treated with PTX and BMX-001 for 24 h and collected for the following CCK-8 test, H2DCFDA ROS staining, qPCR, and antioxidant enzyme activity assays. (**B**) For the in vivo experiment, female C57BL/6 mice were randomly assigned to one of 4 groups in vivo: Sham, PTX, BMX-001, or PTX + BMX-001. Intraperitoneal injections of 8 mg/kg PTX per dose or vehicle solution (ethanol, Cremophor EL, and saline) were given on Days 1, 3, 5, and 7. Subcutaneous injections of 0.5 mg/kg per dose of BMX-001 or PBS were given on Days 1, 3, 5, and 7 and twice a week before the end point at 21 days. Behavioral testing of mice was evaluated using a von Frey filament assay for mechanical allodynia, an acetone drop test for cold allodynia, and a hot plate test for thermal hyperalgesia on the timepoints indicated.

**Figure 2:**
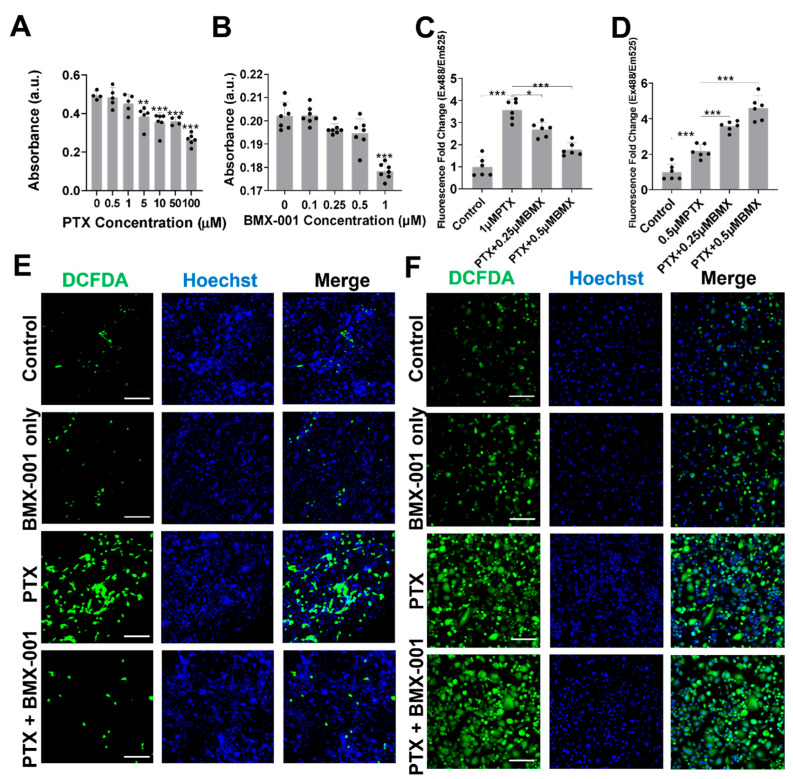
BMX-001 reduced the total ROS elevated by PTX in SGCs but increased ROS in MDA-MB-231 cells in vitro. (**A**,**B**) SGCs were cultured with PTX or BMX in various titrations for 24 h before evaluation of the cell viability by a CCK-8 test (*n* = 4–7). (**C**,**D**) Total ROS levels in SGCs and MDA-MB-231 cells in the Control group, PTX group, PTX with 0.25 μM BMX-001 group, or PTX with 0.5 μM BMX-001 group for 24 h (*n* = 6). (**E**) H2DCFDA fluorescence staining in SGCs after being treated with medium, 0.5 µM BMX-001, 1 µM PTX, or 1 µM PTX + 0.5 µM BMX-001 for 24 h (Scale bar = 100 μm, all figures share same scale bar). (**F**) H2DCFDA fluorescence staining in MDA-MB-231 after being treated with medium, 0.25 µM BMX-001, 0.5 µM PTX, or 0.5 µM PTX + 0.25 µM BMX-001 for 24 h (Scale bar = 200 μm, all panels share same scale bar). * *p* < 0.05, ** *p* < 0.01, *** *p* < 0.001 compared to 0 µM PTX or 0 µM BMX-001.

We started by examining how PTX and BMX-001 influence the behavior of SGCs. SGCs are specialized glial cells located in sensory ganglia, such as DRGs [36]. These cells surround neuronal cell bodies and play vital roles in supporting neurons and regulating neuroimmune interactions [37]. Changes in SGCs and their interactions with DRG neurons have been strongly linked to PTX exposure and are associated with increased pain behaviors observed in CINP assessments [38]. To explore the effects of BMX-001 in vitro, primary SGCs were isolated from mouse DRGs and used as a cellular model, as shown in Figure 1A. The cytotoxic effects of BMX-001 and PTX on SGCs were evaluated using the CCK-8 assay. Neither BMX-001 nor PTX caused significant cell death at concentrations below 0.5 µM and 1 µM (Figure 2A,B). Based on these findings, 0.5 µM BMX-001 and 1 µM PTX were selected as safe concentrations for subsequent in vitro experiments. The elevated ROS production and disruption of the antioxidant defenses caused by chemotherapeutic drugs are major contributors to inflammation and neuronal hyperactivity, which are closely associated with neuropathic pain [39]. To assess BMX-001’s antioxidative properties under PTX-induced oxidative stress, three experimental groups for SGCs and MDA-MB-231 cells, an aggressive human triple-negative breast cancer cell line derived from a patient with triple-negative breast cancer, were established: a control group, a PTX-treated group, and a group treated with both BMX-001 and PTX for one day. For MDA-MB-231, 0.25 µM BMX-001 and 0.5 µM PTX were selected as safe concentrations for subsequent in vitro experiments based on cytotoxicity tests (Figure S1A,B). Total ROS levels in the SGCs were measured using a 2′,7′-dichlorofluorescin diacetate (H2DCFDA) cellular ROS assay kit. ROS levels were significantly elevated in PTX-treated SGCs compared to the controls (Figure 2C). This increased ROS level was markedly reduced with BMX-001 co-treatment for SGCs (Figure 2C and Figure S1C). However, for MDA-MB-231 cells, the BMX-001 and PTX co-treatment group showed significantly elevated total ROS levels, even higher than the PTX-only treatment group (Figure 2D and Figure S1C). Confocal imaging of DCFDA-stained SGCs and MDA-MB-231 cells was performed to further confirm this difference between BMX-001 effects on SGCs and MDA-MB-231cells. The number of SGCs with increased cellular ROS, indicated by green fluorescence, was significantly higher after PTX treatment but significantly decreased when BMX-001 was added, suggesting that BMX-001 acts as an ROS scavenger in this context (Figure 2E). However, in MDA-MB-231 cells, the addition of BMX-001 aggravated the PTX-induced increase in ROS levels (Figure 2F). Overall, these findings demonstrate that BMX-001 exerts antioxidant effects against the PTX treatment in SGCs while showing pro-oxidative effects in MDA-MB-231 cells.

### 2.2. BMX-001 Regulated Inflammation and Antioxidants Differently in SGCs and MDA-MB-231

**Figure 3:**
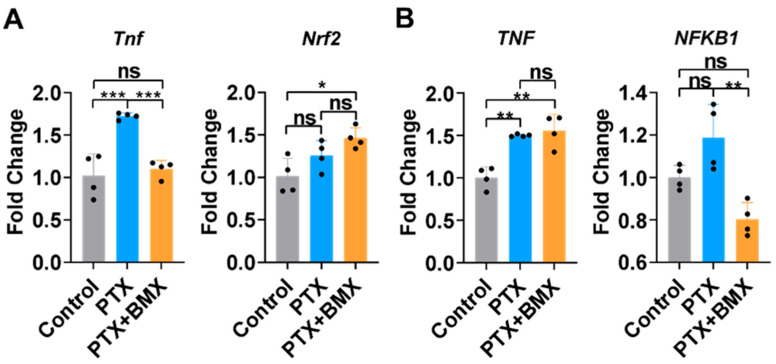
BMX-001 regulated inflammation and antioxidants differently in SGCs and MDA-MB-231. (**A**) Gene expressions of TNF-alpha and Nrf2 in SGCs recorded by qPCR (*n* = 4). (**B**) Gene expressions of TNF-alpha and NF-kB in MDA-MB-231 recorded by qPCR (*n* = 4). * *p* < 0.05, ** *p* < 0.01, *** *p* < 0.001, ns: no significant difference.

Reducing neuronal inflammation is essential for managing CINP progression [40]. To explore the potential antioxidant and anti-inflammatory properties of BMX-001, the SGCs and MDA-MB-231 cells treated with PTX and BMX-001 were harvested and analyzed for gene expression of inflammatory and antioxidant markers by using qPCR (Figure 3A,B). For SGCs, the expression of tumor necrosis factor alpha (TNF-α), a well-studied pro-inflammatory cytokine, was significantly upregulated following PTX treatment compared to controls (Figure 3A). However, this elevated TNF-α expression was significantly suppressed by BMX-001 co-treatment with PTX (Figure 3A). In MDA-MB-231 cells, PTX treatment led to a marked increase in TNF-α expression, but the TNF-α expression was not reduced but preserved in the presence of BMX-001 in MDA-MB-231 cells (Figure 3B).

We also examined Nrf2, a central transcription factor involved in regulating antioxidant and anti-inflammatory responses. Although PTX treatment did not affect Nrf2 expression in SGC, BMX-001 treatment led to a significant increase in Nrf2 levels compared to the control group, suggesting an enhanced antioxidant response to PTX stimulation (Figure 3A). NF-kB, a key transcription factor in regulating inflammatory cytokine production, was significantly downregulated in the BMX-001 and PTX co-treatment group compared to the controls in MDA-MB-231 cells (Figure 3B).

### 2.3. BMX-001 Regulated Antioxidant Enzyme Activity Differently in SGCs and MDA-MB-231

**Figure 4:**
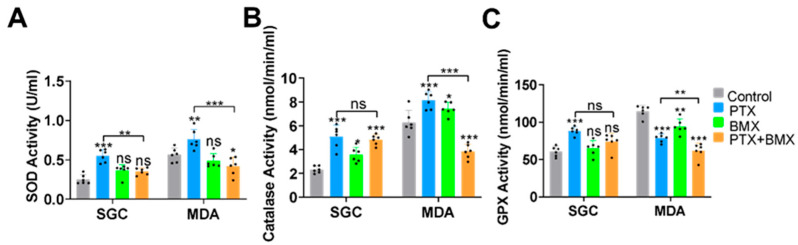
BMX-001 regulated antioxidant enzyme activity differently in SGCs and MDA-MB-231. SOD (**A**), catalase (**B**), and GPx (**C**) enzyme activity in SGCs and MDA-MB-231 cells after the PTX and BMX-001 treatments (SGCs treatments: 1 μM PTX, 0.5 μM BMX-001; MDA-MB-231 treatments: 0.5 μM PTX, 0.25 μM BMX-001) (*n* = 6). * *p* < 0.05, ** *p* < 0.01, *** *p* < 0.001 compared with the Control group, ns: no significant difference.

Cellular antioxidant enzymes play critical roles in mitigating PTX-induced oxidative stress [41]. To further investigate the potential mechanisms underlying the differential effects of BMX-001 in SGCs and MDA-MB-231 cells, the activities of three major antioxidant enzymes, i.e., superoxide dismutase (SOD), catalase, and glutathione peroxidase (GPx), were measured in both cell types after one-day treatment with BMX-001 and PTX (Figure 4A–C). In SGCs, PTX significantly increased activity of the three antioxidant enzymes (Figure 4A–C). The BMX-001 treatment alone significantly increased catalase activity but had no effect on SOD and GPx activities (Figure 4A–C). Co-treatment with BMX-001 and PTX significantly reduced SOD activity compared to PTX alone (Figure 4A), while catalase and GPx activities remained unchanged (Figure 4B,C). In MDA-MB-231 cells, PTX similarly enhanced the activity of SOD and catalase without affecting GPx, suggesting elevated ROS and H_2_O_2_ accumulation under stress conditions (Figure 4A–C). Interestingly, co-treatment with BMX-001 and PTX significantly decreased the activities of SOD, catalase, and GPx compared to PTX treatment alone, indicating a potential disruption of the antioxidant defense system overwhelmed by high intracellular ROS stress (Figure 4A–C). BMX-001 treatment alone significantly increased catalase activity, but it reduced GPx activity (Figure 4A–C). Overall, in SGCs, BMX-001 mitigates PTX-induced inflammation and preserves antioxidant enzyme activity. In contrast, in MDA-MB-231 cells, BMX-001 appears to compromise the antioxidant defense system under PTX-induced stress, suggesting a cell-specific modulation of anti-inflammation and antioxidant responses by BMX-001.

### 2.4. BMX-001 Alleviated Mechanical and Thermal Allodynia in a Mouse Model of CINP

**Figure 5:**
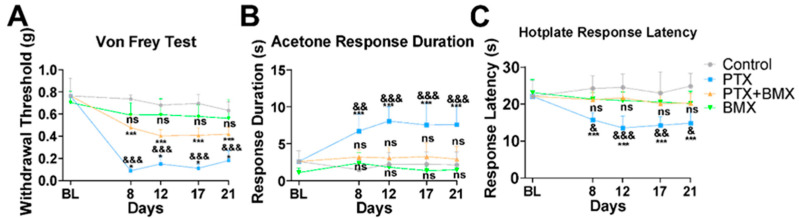
BMX-001 alleviated mechanical and thermal allodynia in a mouse model of CINP. (**A**–**C**) Behavioral test results of the in vivo CINP model mice described in Figure 1B measured by a von Frey test, acetone drop test, and hot plate test (*n* = 7). PTX: 8 mg/kg per dose (32 mg/kg for accumulated four doses in one week); BMX-001: 0.5 mg/kg per dose (4 mg/kg for accumulated eight doses in three weeks); PTX + BMX: PTX and BMX treatments combined. Statistical significance for Control vs. PTX Control vs. BMX-001, and Control vs. PTX + BMX-001: * *p* < 0.05, *** *p* <0.001, ns: no significant difference; PTX vs. PTX + BMX-001: & *p* < 0.05, && *p* < 0.01, &&& *p* < 0.001.

Chemotherapeutic agents like PTX are well recognized for their detrimental effects on peripheral nerves, especially in distal regions of the body where nerve fibers are more susceptible to damage. This often results in debilitating pain and sensory nerve function loss, which leads to numbness or burning primarily in the hands and feet [42,43]. To investigate the effects of BMX-001 on pain relief in vivo, we established a 3-week CINP mouse model by administering PTX intraperitoneally. BMX-001 was administered subcutaneously every other day during the first week and twice weekly during the following two weeks, as illustrated in Figure 1B. Pain sensitivity assessments, including mechanical, cold, and hot stimuli, were conducted using the von Frey filament test, the acetone spray test, and the hotplate test, respectively, after PTX and BMX-001 administration. Following repeated PTX injections, the mice exhibited mechanical allodynia starting on day 8, which persisted over the subsequent weeks (Figure 5A). BMX-001 treatment alone did not affect the mechanical sensitivity of the mice, but co-treatment with PTX and BMX-001 significantly alleviated mechanical allodynia in CINP mice (Figure 5A). Similarly, acetone and hotplate test results showed that PTX-treated mice had a delayed response time to cold stimulation (Figure 5B) and reduced withdrawal latency on the hotplate (Figure 5C), indicating exacerbated thermal hyperalgesia. BMX-001 alone did not induce significant thermal sensitization; however, co-treatment with PTX and BMX-001 effectively mitigated PTX-induced thermal allodynia (Figure 5A–C). These findings highlight BMX-001’s efficacy in alleviating sensory symptoms of CINP.

### 2.5. BMX-001 Ameliorated Inflammation, ROS, and Hyposensitivity in Peripheral Nerves of Mice with CINP

**Figure 6:**
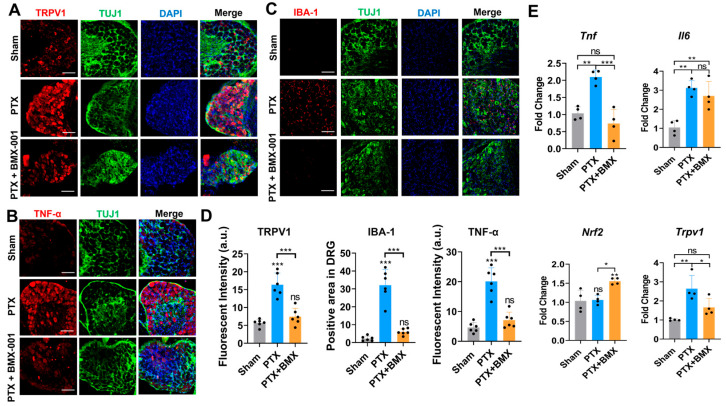
BMX-001 ameliorated inflammation, ROS, and hyposensitivity in peripheral nerves of mice with CINP. (**A**–**C**) Immunostaining results of TRPV1, IBA-1, and TNF-alpha in the isolated mouse DRGs from the in vivo study mice (Scale bar = 100 μm, all panels share the same scale bar). (**D**) Quantification of the fluorescent signal intensity of the TRPV1 in (**A**), positive area of IBA-1 in (**B**), and fluorescent signal intensity of TNF-alpha in (**C**) (*n* = 6). (**E**) Gene expressions of TNF-alpha, IL-6, Nrf2, and TRPV1 in the isolated mouse DRGs from the in vivo study mice recorded by qPCR (*n* = 4). * *p* < 0.05, ** *p* < 0.01, *** *p* < 0.001, ns: no significant difference compared with Sham group.

To explore the role of BMX-001 in mitigating allodynia, we performed IF staining on DRGs isolated from mice across the three experimental groups (Sham, PTX, and PTX + BMX-001). After PTX treatment, the expression of transient receptor potential vanilloid 1 (TRPV1), a key marker involved in pain and thermal sensitivity, was markedly elevated in the DRGs compared to the Sham group, indicating heightened pain sensitivity induced by PTX (Figure 6A). However, BMX-001 treatment significantly reduced this increased TRPV1 expression, which is consistent with the behavioral data showing BMX-001’s pain-suppressing effects. (Figure 6A). We further assessed chemotherapy-induced inflammation by staining DRGs for TNF-α in CINP mice. PTX administration led to a notable increase in the TNF-α signal, indicating the activated inflammation in the peripheral nervous system (Figure 6B). This effect was reversed by BMX-001 treatment, suggesting its anti-inflammatory action (Figure 6B). This increased TNF-α expression was also observed in the isolated sciatic nerves from the CINP mice (Figure S2). Chemotherapy-induced inflammation often triggers immune cell infiltration, especially macrophages, into the damaged nerves and tissues [44]. To evaluate this inflammatory response, we stained DRGs for ionized calcium-binding adaptor molecule 1 (IBA-1), a pan-inflammatory marker specifically expressed in macrophages and microglia. BMX-001 treatment inhibited the PTX-induced increase in IBA-1 expression in DRGs, suggesting that local macrophage accumulation was successfully prevented (Figure 6C). These conclusions were also confirmed by the quantitative analysis of the TRPV1, IBA-1, and TNF-α marker IF signal intensity in Figure 6A–C, which revealed significant differences among the groups (Figure 6D).

To validate the anti-inflammatory and antioxidant properties of BMX-001, we performed qPCR to analyze the expressions of related markers in DRGs isolated from CINP mice. As observed earlier in the in vitro results, PTX treatment significantly increased the expression of pro-inflammatory cytokines, such as TNF-α and IL-6 (Figure 6E). BMX-001 treatment significantly prevented this TNF-α upregulation. In line with previous in vitro qPCR results, Nrf2 expression was not affected by PTX alone but was notably upregulated by BMX-001 (Figure 6E). Additionally, TRPV1 expression, elevated by PTX treatment, was markedly suppressed by BMX-001 co-treatment (Figure 6E). These results demonstrate the neuroprotective effects of BMX-001 against PTX-induced hypersensitivity, inflammation, and oxidative stress.

## 3. Discussion

BMX-001 has been recognized for its protection of normal tissue and its mitochondrial SOD2-mimicking role in scavenging ROS by reducing superoxide in radiation therapy in preclinical studies [28]. The role of BMX-001 in protecting cells against radiation and chemotherapy has been well reported across various diseases, like high-grade gliomas, head and neck cancers, and non-small cell lung cancers [26,34,45,46]. It was also documented that it protects cardiomyocytes from hypoxia-induced oxidative stress under ischemia–reperfusion injury, drawing increased clinical interest [47]. Our study also demonstrated that BMX-001 effectively managed intracellular oxidative damage and modulated inflammatory pathways. BMX-001 specifically reduced PTX-induced ROS levels in SGCs, which act as sensors of ROS and nerve injury surrounding neuron somas and serve as key regulators of the neuronal microenvironment in the DRGs [38]. This effect aligns with the SOD-mimicking properties of its manganese porphyrin structure [48].

BMX-001’s effect on cancer cells has been reported to enhance sensitivity to radiation or chemotherapy, rather than protecting tumors [26]. In alignment with these previous studies, our results demonstrated BMX-001’s dual function in stabilizing SGCs while suppressing MDA-MB-231 during PTX therapy. In vitro, BMX-001 reduced PTX-induced ROS levels in SGCs but increased ROS levels in MDA-MB-231 cells. This differential regulation of inflammation and antioxidant activity aligned with data showing that BMX-001 enhanced tumor cytotoxicity in preclinical models of brain, colorectal, and breast malignancies [25]. In ovarian cancer models, co-treatment with BMX-001 and PTX significantly reduced the cell viability of CAOV2 cells compared to PTX alone and downregulated the expression of BCL2, NF-κB, and IL-1β [49].

The safety of BMX-001 in normal tissue has been evaluated and confirmed by Gad et al. in nonclinical toxicokinetic and systemic toxicity studies in both mice and non-human primates. BMX-001, administered by subcutaneous injection, showed no observed adverse effects or organ toxicities below the initial loading/maintenance dose of 12/2 mg/kg in mice and 6/2 mg/kg in monkeys [50]. In a phase I clinical trial of newly diagnosed high-grade glioma patients, subcutaneous BMX-001 injections of one loading dose of 28 mg before radiation and 2 times/week of 14 mg doses after radiation for 8 weeks in 15 patients aged from 19 to 80 years were tolerated well. The only grade 3 toxicities attributable to BMX-001 were transient sinus tachycardia (*n* = 1) and hypotension (*n* = 1); Although a grade 1 injection-site reaction (*n* = 7) was observed, no evidence of bone marrow suppression or organ toxicity was observed [51].

However, the mechanism underlying BMX-001’s differential effects in normal cells and cancer cells is complex and largely unclear. Previous studies have reported that BMX-001 levels are up to tenfold higher in 4T1 mouse breast cancer tissues compared to normal tissues, suggesting preferential accumulation in tumor tissues [52]. Another possibility is that BMX-001, as a potent enzymatic redox agent, disrupts redox balance unfavorably in tumors. It catalyzes the conversion of superoxide into hydrogen peroxide (H_2_O_2_), which is highly cytotoxic to tumor cells due to their already elevated baseline levels of ROS and H_2_O_2_ [32]. This mechanism is crucial in enhancing the efficacy of radiation and chemotherapeutic agents like PTX, both of which generate substantial superoxide [53]. In a preclinical in vitro breast cancer model, PTX-induced bystander cancer cell killing was amplified by SOD and inhibited by catalase, indicating that the effect was mediated by H_2_O_2_ [54]. Consistent with this mechanism, our data showed that MDA-MB-231 cells exhibit a higher baseline of activity of antioxidant enzymes (SOD, catalase, and GPx) compared to SGCs. Although PTX alone increased the activity of all three antioxidant enzymes in both cell types as a response to oxidative stress, co-treatment with BMX-001 maintained the normal activity of SOD and GPx in SGCs but significantly reduced the activity of all three enzymes in MDA-MB-231, leading to redox imbalance and H_2_O_2_-induced cytotoxicity. Notably, GPx is more sensitive to H_2_O_2_ and responds rapidly to oxidative stress but is usually the first to become inactivated under prolonged ROS exposure [55]. Catalase, by contrast, responds more slowly but may be upregulated by sustained oxidative stress to serve as a longer-term detoxification mechanism [56]. Consistent with the difference mentioned above, BMX alone increased catalase activity while reducing GPx activity in MDA-MB-231 cells. This may reflect an initial upregulation followed by depletion of GPx and a compensatory increase in catalase expression. In SGCs, BMX-001 increased catalase activity but left GPx levels unchanged, suggesting ROS levels were elevated but remained under control—possibly due to recoverable GPx activity. The persistent catalase elevation may be driven by Nrf2 pathway activation, providing long-term detoxification, which warrants further investigation into the temporal dynamics of ROS and redox regulation by BMX-001.

Importantly, BMX-001 likely promotes mild redox modulation that stabilizes Nrf2 and suppresses NF-κB in normal cells, like SGCs, without benefiting cancer cells, where the Nrf2 pathway may already be dysregulated [25]. BMX-001 has also been shown to oxidize the p50 subunit of NF-κB, thereby suppressing tumor progression while protecting normal cells from chemoradiation-induced damage [57]. This is consistent with our qPCR results, showing upregulation of Nrf2 in SGCs and downregulation of NF-κB in MDA-MB-231 cells. Additionally, BMX-001 reduced TNF-α production in SGCs but not in MDA-MB-231 cells, further supporting its dual role as an antioxidant in normal cells and a pro-oxidant in cancer cells.

However, certain limitations exist when interpreting the findings in the current study. We utilized a short-term PTX-induced CINP model, which may not capture the chronic and persistent conditions of neuropathy in cancer patients. Only a single breast cancer cell line, MDA-MB-231, was examined in this study, which limited the generalizability of the findings. The potential tumor heterogeneity may also influence the redox-modulating effects of BMX-001. Long-term studies, more cancer models, and a detailed pharmacokinetic/pharmacodynamic evaluation of BMX-001 will be required for crossing the translational gap between murine models and clinical applications.

To evaluate the specific role and efficacy in neuroinflammation and CINP in vivo, we developed a mouse CINP model using i.p. injection of PTX to assess the CINP-related hypersensitization behaviors and inflammation-related changes. In the DRGs isolated from the CINP mice, BMX-001 treatment prevented PTX-induced neuroinflammation, as exhibited by a reduced expression of pro-inflammatory cytokines TNF-α and IL-6, as measured by qPCR analysis. Additionally, IF staining of IBA-1 revealed that BMX-001 attenuated PTX-induced macrophage infiltration into peripheral nerves and DRGs, limiting the accumulation of pro-inflammatory mediators and ROS. We also demonstrated that BMX-001 prevented the hyperactivation of sensory neurons and nociceptors by controlling the overexpression of TRPV1. These findings aligned with our behavioral assays, including von Frey, acetone, and hotplate tests, confirming BMX-001’s efficacy in alleviating mechanical and thermal allodynia.

Although our in vivo analyses in this study focused on the DRG, our in vitro experiments used isolated SGCs rather than primary DRG neurons due to their pivotal role in maintaining the neuronal microenvironment and active participation in the pathogenesis of CINP. By targeting SGCs, we aimed to elucidate the glial-specific effects of BMX-001 under oxidative and inflammatory stress to provide mechanistic insights complementary to our in vivo behavioral results. We plan to incorporate the direct in vitro investigation of DRG neurons in future work.

**Figure 7:**
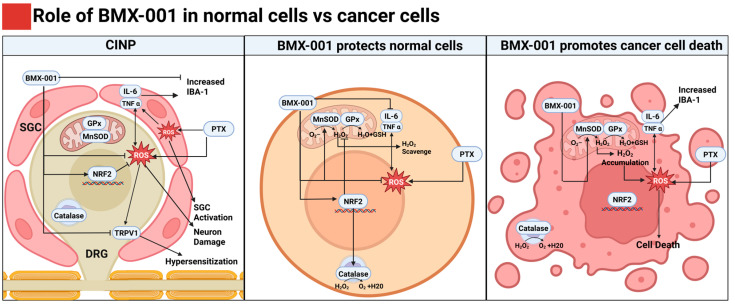
BMX-001’s potential roles in PTX-induced CINP and its differential effects on normal cells versus MDA-MB-231 cancer cells. In CINP, PTX induces the expression of pro-inflammatory cytokines, like IL-6 and TNF-α, in SGCs and TRPV1 sensitization in DRG through ROS accumulation, leading to glial activation, recruitment of immune cells, neuronal damage, and thus hypersensitization. In normal cells, like SGCs and neuronal cells, BMX-001 reduces the total ROS level and TNF-α expression through quick scavenging superoxide, upregulation of Nrf2, and enhanced activity of catalase, which protect the DRG neurons from PTX-induced CINP. Meanwhile, in cancer cells like MDA-MB-231, BMX-001 enhances excess oxidative stress by promoting H_2_O_2_ accumulation, which leads to inefficient ROS and H_2_O_2_ clearance because of the exhausted antioxidant enzymes, like MnSOD, catalase, and GPx, and thus the enhanced cell death and stimulated immune cell recruitment.

Overall, this study provides evidence supporting the substantial translational potential of BMX-001 in CINP treatment, as illustrated in Figure 7. Its favorable safety profile, demonstrated in multiple oncology trials, and its cell-specific antioxidant and anti-inflammatory role make it an attractive adjunct for chemotherapy drugs [50]. By mitigating ROS and inflammation in normal tissue while preserving or enhancing anti-tumor activity, BMX-001 holds promise for clinical application in preventing CINP for patients undergoing PTX-based therapies. However, the pharmacokinetics of BMX-001 in combination with PTX remain incompletely understood, particularly its biodistribution and interaction dynamics in systemic and peripheral tissues. The long-term effects of BMX-001 on chronic CINP models also require exploration since CINP often persists for months or years in clinical settings. Future research will also be performed to examine BMX-001’s broader effects on the components involved in pain processing, other than SGCs, sciatic nerves, and DRGs. Further investigation into the detailed mechanism of BMX-001’s cell-specific effect on normal cells and cancer cells is also in need to achieve a better understanding of its neuroprotective mechanisms.

## 4. Materials and Methods

### 4.1. Mouse SGCs Isolation and Culture

SGCs were isolated from DRGs obtained from 2 to 4-week-old mice with a C57BL/6 background using our previously described method [58], under a protocol approved by the Institutional Animal Care and Use Committee (IACUC) of the University of Nebraska Medical Center (UNMC). Following euthanasia, the L4-5 DRGs were dissected and kept on ice in HBSS (Gibco, Thermo Fisher Scientific, Waltham, MA, USA). Subsequently, the DRGs were transferred to a solution containing HBSS and papain prewarmed to 37 °C and incubated for 20 min. The tissues were then centrifuged at 400 rpm for 3 min, and the supernatant was gently discarded. After rinsing with 3 mL of prewarmed HBSS, the DRGs were incubated with a mixture of collagenase, dispase, and TrypLE (1:1:1, 500 μL each; Gibco) at 37 °C for 20 min. The cells were centrifuged again at 500 rpm for 5 min, resuspended in standard culture medium, and plated in poly-D-lysine-coated flasks (Thermo Fisher Scientific, Waltham, MA, USA). The cells were cultured in DMEM (HyClone, Cytiva, Logan, UT, USA) supplemented with 10% FBS (Gibco) and 1% penicillin-streptomycin (P/S, Invitrogen, Thermo Fisher Scientific, Waltham, MA, USA) at 37 °C in a 5% CO_2_ environment. Characterization of SGCs was carried out using markers such as glial fibrillary acidic protein (GFAP), glutamine synthetase, and S100B.

### 4.2. In Vitro Cytotoxicity Assay for PTX and BMX-001

The cytotoxic effects of PTX and BMX-001 on SGCs and MDA-MB-231 were assessed using a Cell Counting Kit 8 (CCK-8; Abcam, Cambridge, MA, USA). SGCs (30,000 cells/cm^2^) and MDA-MB-231 (90,000 cells/cm^2^) were seeded into 96-well plates and cultured overnight in DMEM supplemented with 10% FBS and 1% P/S. To evaluate PTX toxicity, the cells were treated with PTX at concentrations of 0, 0.5, 1, 5, 10, 50, and 100 μM for 24 h. Similarly, for BMX-001, SGCs and MDA-MB-231 were exposed to 0, 0.1, 0.25, 0.5, 1, and 2 μM for the same duration. After treatment, the cells were incubated with CCK-8 solution for 1 h, and absorbance at 460 nm was measured using a microplate reader (Synergy H1, BioTek Instruments, Inc., Winooski, VT, USA).

### 4.3. In Vitro ROS Evaluation

Intracellular ROS levels were measured using a 2′,7′-dichlorofluorescin diacetate (H2DCFDA) Cellular ROS Assay Kit (Abcam). SGCs and MDA-MB-231 were seeded in 96-well plates and cultured overnight in DMEM with 10% FBS and 1% P/S. SGC cells were treated with either medium alone, 1 μM PTX, 1 μM PTX + 0.25 μM BMX-001, or 1 μM PTX + 0.5 μM BMX-001 for 24 h. MDA-MB-231 cells were treated with either medium alone, 0.5 μM PTX in medium, 0.5 μM PTX +0.25 μM BMX-001, or 0.5 μM PTX + 0.5 μM BMX-001 for 24 h. Following treatment, the cells were incubated with an H2DCFDA solution for 1 h, and the fluorescence was measured at 460 nm using a microplate reader. Confocal laser scanning microscopy (Zeiss LSM 710; Carl Zeiss Microscopy GmbH, Jena, Germany) was also employed to capture images at Ex488/Em525.

### 4.4. In Vitro SOD, Catalase, and GPx Activity Assay

The activities of SOD, catalase, and GPx in SGCs and MDA-MB-231 were tested by using SOD, catalase, and GPx Assay Kit (Cayman Chem, Ann Arbor, MI, USA). Briefly, SGCs and MDA-MB-231 were seeded in 96-well plates and cultured overnight in DMEM with 10% FBS and 1% P/S. SGCs were treated with either medium alone, 1 μM PTX, 0.5 μM BMX-001, or 1 μM PTX + 0.5 μM BMX-001 for 24 h. MDA-MB-231 cells were treated with either medium alone, 0.5 μM PTX, 0.25 μM BMX-001, or 0.5 μM PTX + 0.25 μM BMX-001 for 24 h. Then the cells were collected by centrifugation at 4 °C and homogenized in cold buffer. The collected supernatant was then used to react with the assay buffer in 96-well plates before reading the absorbance using a microplate reader (Synergy H1, BioTek).

### 4.5. CINP Mouse Model and Treatments

Animal experiments were conducted under protocols approved by the IACUC at UNMC. A total of 28 female C57BL/6 mice aged 56–62 days (18–21 g) were purchased from Charles River Laboratories and randomly divided into four groups (*n* = 7/group): (1) Sham group treated with vehicle solution (ethanol, Cremophor EL, and saline at a 1:1:8 ratio); (2) PTX only group (8 mg/kg per dose, 0.8 mg/mL PTX solution); (3) BMX-001 only group (0.5 mg/kg); and (4) BMX-001 treatment group treated with PTX (8 mg/kg per dose) and BMX-001 (0.5 mg/kg per dose). The dose for PTX and BMX-001 was selected based on previously published studies [34,50,59,60,61,62,63]. Baseline behavior tests were performed on Day 0, and PTX or the vehicle was administered intraperitoneally on Days 1, 3, 5, and 7. BMX-001 was subcutaneously injected on Days 1, 3, 5, and 7 in the first week and biweekly in the following two weeks (Days 9, 11, 15, and 18). On Day 21, the mice were euthanized, and the sciatic nerves and DRGs were harvested for analysis.

### 4.6. Behavioral Tests

Mechanical allodynia, cold sensitivity, and heat sensitivity were evaluated using von Frey, acetone, and hotplate tests, respectively [64]. The mice were placed in boxes atop a raised metal grid for acclimatization before testing. Sensitivity to tactile stimuli was assessed using von Frey filaments (0.008–2.0 g, Aesthesio^®^, Stoelting Co., Wood Dale, IL, USA). Withdrawal responses, including paw shaking and licking, were recorded across five trials per mouse, with 15 min intervals. Cold sensitivity was measured by applying 50 μL of acetone to the hind paws and recording the duration of responses within 60 s. Heat sensitivity was assessed by placing mice on a hotplate and recording the latency of paw withdrawal responses.

### 4.7. Immunofluorescence (IF) Imaging

Tissues from sciatic nerves and DRGs were fixed in 4% PFA, cryoprotected in 30% sucrose, and embedded in OCT before cryosectioning. Sections were treated with methanol, washed, and blocked with 3% goat serum and 0.4% Triton X-100 (Sigma-Aldrich, St. Louis, MO, USA). Primary antibodies against TNF-alpha, TRPV1, IBA-1, and TUJ1 were applied overnight at 4 °C. After washing, secondary antibodies (Alexa Fluor 488/568; Thermo Fisher Scientific, Waltham, MA, USA) and DAPI were used for staining. Fluorescence imaging was performed with a Zeiss LSM 710 microscope. For in vitro dihydroethidium DHE staining, SGCs were treated with 10 μM DHE for 1 h at 37 °C before imaging.

### 4.8. RNA Isolation and Quantitative PCR

Total RNA was extracted from SGCs and DRGs using a RNeasy Mini Kit (QIAGEN, Hilden, Germany) and reverse transcribed into cDNA with a Bio-Rad iScript cDNA Kit (Bio-Rad Laboratories, Hercules, CA, USA). Quantitative PCR was conducted using the StepOnePlus PCR System (Thermo Fisher Scientific) and SYBR Green Supermix (Bio-Rad). Target gene expression levels were analyzed using the comparative Ct (2^−ΔΔCt^) method.

### 4.9. Statistical Analysis

Descriptive statistics, including mean, standard deviation, median, first quartile, third quartile, and box plot visualizations, were used to summarize the data. Data are presented as mean ± SEM. Student’s *t*-tests were conducted for comparisons between two groups, and one-way ANOVA followed by Tukey’s post hoc tests were used for multiple-group comparisons. *p* < 0.05 was considered statistically significant. Longitudinal data analysis, including repeated-measures ANOVA, was conducted to assess changes over time. Statistical significance was defined as * *p* < 0.05, ** *p* < 0.01, and *** *p* < 0.001. All analyses were performed using GraphPad Prism version 10 and SAS version 9.4.

## 5. Conclusions

In this preclinical study, we evaluated BMX-001 as a potential therapeutic adjunct for managing CINP. By targeting oxidative stress and neuroinflammation, BMX-001 offers a mechanism-driven approach to preserve chemotherapeutic efficacy while mitigating its neurotoxic side effects in our in vitro and in vivo models. These preclinical findings support BMX-001’s dual role in protecting healthy tissues while enhancing tumor-killing effects, which suggests BMX-001 may serve as a valuable addition to PTX-based regimens and potentially other neurotoxic chemotherapies. However, further studies are necessary to confirm its safety and translational potential.

## Data Availability

The data that support the findings of this study are available from the corresponding authors upon reasonable request.

## Supplementary Materials

The following supporting information can be downloaded at: https://www.mdpi.com/article/10.3390/ph18081159/s1, Figure S1: (A,B) The cytotoxicity assay results of MDA-MB-231 after being treated with BMX-001 or PTX in different titrations for 24 h by a CCK-8 test (*n* = 8). * *p* < 0.05, ** *p* <0.01, *** *p* <0.001, ns: no significant difference. (C) Total ROS levels in SGCs and MDA-MB-231 cells in the Control group, PTX group, PTX with 0.25 μM BMX-001 group, or PTX with 0.5 μM BMX-001 group for 24 h by H2DCFDA staining assay (*n* = 6). Statistical significance for each group vs. Control group for SGCs: * *p* < 0.05, ** *p* < 0.01, *** *p* < 0.001, ns: no significant difference; statistical significance for each group vs. Control group for MDA-MB-231 cells: & *p* < 0.05, && *p* < 0.01, && *p* < 0.001.; Figure S2: (A) Immunostaining results of TNF-alpha in the isolated mouse sciatic nerve from the in vivo study mice (Scale bar = 50 μm).

## Abbreviations

The following abbreviations are used in this manuscript:

CINP	Chemotherapy-induced neuropathic pain
DRG	Dorsal root ganglion
GFAP	Glial fibrillary acidic protein
GPx	Glutathione peroxidase
H_2_O_2_	Hydrogen peroxide
H2DCFDA	2′,7′-dichlorofluorescin diacetate
IBA	Ionized calcium-binding adaptor molecule
IF	Immunofluorescence
Nrf2	Nuclear factor erythroid 2-related factor 2
PTX	Paclitaxel
ROS	Reactive oxygen species
SGC	Satellite glial cell
SOD	Superoxide dismutase
TNF	Tumor necrosis factor
TRPV	Transient receptor potential vanilloid
