# Correction: Pan et al. Therapeutic Potential of BMX-001 for Preventing Chemotherapy-Induced Peripheral Neuropathic Pain. *Pharmaceuticals* 2025, *18*, 1159

**DOI:** 10.3390/ph19030351

**Published:** 2026-02-25

**Authors:** Tianshu Pan, Olawale A. Alimi, Bo Liu, Mena A. Krishnan, Mitchell Kuss, Wei Shi, Jairam Krishnamurthy, Jianghu James Dong, Rebecca E. Oberley-Deegan, Bin Duan

**Affiliations:** 1 Mary & Dick Holland Regenerative Medicine Program, University of Nebraska Medical Center, Omaha, NE 68198, USA; tpan@unmc.edu (T.P.); oalimi@unmc.edu (O.A.A.); bo.liu@unmc.edu (B.L.); mekrishnan@unmc.edu (M.A.K.); mitchell.kuss@unmc.edu (M.K.); weshi@unmc.edu (W.S.); 2 Division of Cardiology, Department of Internal Medicine, University of Nebraska Medical Center, Omaha, NE 68198, USA; 3 Department of Biochemistry and Molecular Biology, University of Nebraska Medical Center, Omaha, NE 68198, USA; 4 Division of Oncology & Hematology, Department of Internal Medicine, University of Nebraska Medical Center, Omaha, NE 68198, USA; jairam.krishnamurthy@hsc.utah.edu; 5 Department of Biostatistics, University of Nebraska Medical Center, Omaha, NE 68198, USA; jianghu.dong@unmc.edu; 6 Department of Surgery, University of Nebraska Medical Center, Omaha, NE 68198, USA; 7 Department of Mechanical and Materials Engineering, University of Nebraska-Lincoln, Lincoln, NE 68588, USA

## Citation Correction

In the original publication [1], Section “3. Discussion”. Original sentence: “In ovarian cancer models, co-treatment with BMX-001 and PTX significantly reduced the cell viability of CAOV2 cells compared to PTX alone and downregulated the expression of BCL2, NF-κB, and IL-1β [49].” 

Original citation for [49] is “Batinic-Haberle, I.; Tovmasyan, A.; Veade, A.; Spasojevic, I.; Reihani, S.; Secord, A. Mn porphyrin/ascorbate sensitizes serous epithelial ovarian cancer to chemotherapy. *Free. Radic. Biol. Med.* **2018**, *128*, S74–S75.” It has been correctly replaced with [2].

The authors state that the scientific conclusions are unaffected. This correction was approved by the Academic Editor. The original publication has also been updated.
