# Supplementary material for: Therapeutic Potential of BMX-001 for Preventing Chemotherapy-Induced Peripheral Neuropathic Pain
Source: Pharmaceuticals (Basel). 2025 Aug 5;18(8):1159. doi: 10.3390/ph18081159 (PMC12389141; doi:10.3390/ph18081159)
Supplement: Supplementary file 1 [file pharmaceuticals-18-01159-s001.zip › pharmaceuticals-3779114-supplementary.pdf]

Supplementary Materials:

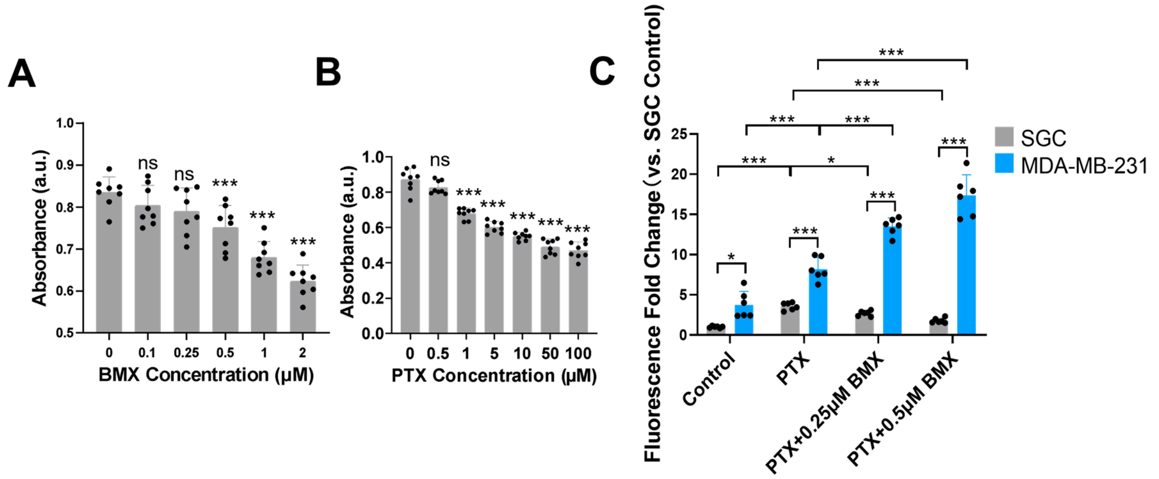

Figure S1. (A-B) The cytotoxicity assay results of MDA-MB-231 after being treated with BMX-001 or PTX in different titrations for 24h by CCK-8 test as described in the Method section (n=8). *p<0.05, **p <0.01, ***p <0.001, ns: no significant difference (compared to 0μM BMX-001 or 0μM PTX). (C) Total ROS levels in SGCs and in MDA-MB-231 cells in the Control group, PTX group, PTX with 0.25μM BMX-001 group or PTX with 0.5μM BMX-001 group for 24h by H2DCFDA staining assay as described in the Method section (fluorescence data from the two cell types were normalized to the same cell number) (n=6). *p<0.05, **p <0.01, ***p <0.001.

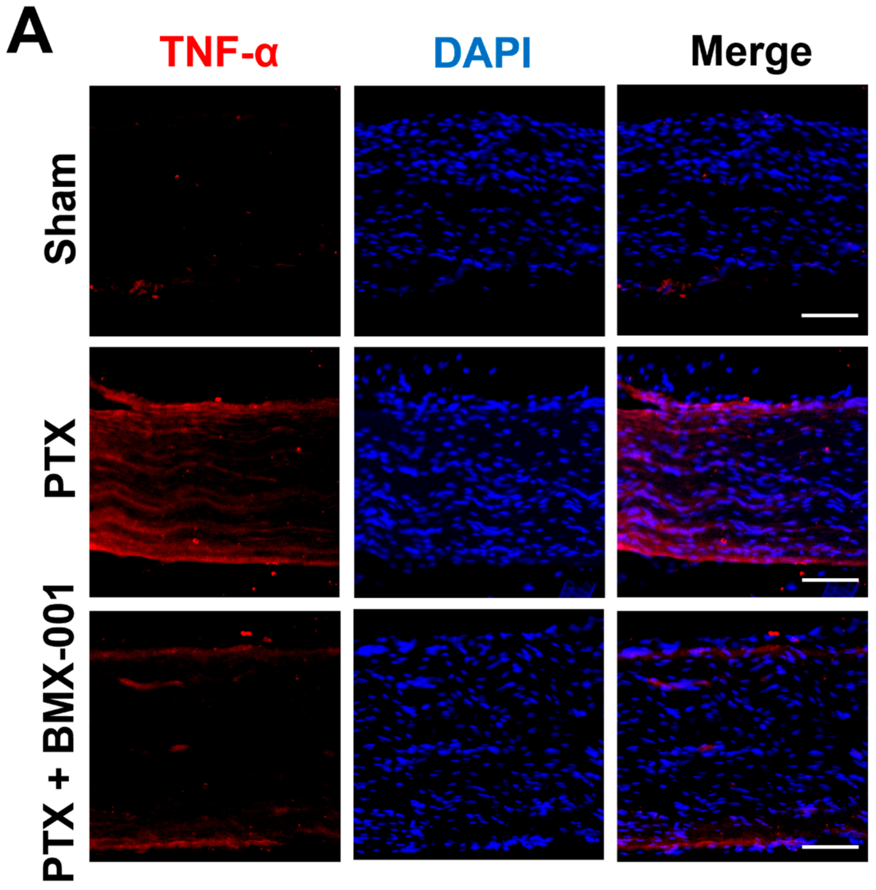

Figure S2. (A) Immunostaining results of TNF-alpha in the isolated mouse sciatic nerve from the in vivo study mice. On Day 21, the CINP mice were euthanized, and their sciatic nerves were harvested

for immunostaining analysis as indicated in the Method section. (Scale bar = 50 μm, all panels share same scale bar).
